# Identification of Biological Properties of Intralymphatic Tumor Related to the Development of Lymph Node Metastasis in Lung Adenocarcinoma

**DOI:** 10.1371/journal.pone.0083537

**Published:** 2013-12-23

**Authors:** Keisuke Kirita, Genichiro Ishii, Rie Matsuwaki, Yuki Matsumura, Shigeki Umemura, Shingo Matsumoto, Kiyotaka Yoh, Seiji Niho, Koichi Goto, Hironobu Ohmatsu, Yuichiro Ohe, Kanji Nagai, Atsushi Ochiai

**Affiliations:** 1 Pathology Division, Research Center for Innovative Oncology, National Cancer Center Hospital East, Chiba, Japan; 2 Division of Thoracic Oncology, National Cancer Center Hospital East, Chiba, Japan; 3 Division of Thoracic Surgery, National Cancer Center Hospital East, Chiba, Japan; 4 Juntendo University Graduate School of Medicine, Tokyo, Japan; University of Nebraska Medical Center, United States of America

## Abstract

**Background:**

Intralymphatic tumors in the extratumoral area are considered to represent the preceding phase of lymph node metastasis. The aim of this study was to clarify the biological properties of intralymphatic tumors susceptible to the development of lymph node metastasis, with special reference to the expression of cancer initiating/stem cell (CIC/CSC) related markers in cancer cells and the number of infiltrating stromal cells.

**Material and Methods:**

Primary lung adenocarcinomas with lymphatic permeation in the extratumoral area were retrospectively examined (n = 107). We examined the expression levels of CIC/CSC related markers including ALDH1, OCT4, NANOG, SOX2 and Caveolin-1 in the intralymphatic cancer cells to evaluate their relationship to lymph node metastasis. Moreover, the number of infiltrating stromal cells expressing CD34, α-smooth muscle actin, and CD204 were also evaluated.

**Results:**

Among the intralymphatic tissues, low ALDH1 expression in cancer cells, high SOX2 expression in cancer cells, and a high number of CD204(+) macrophages were independent predictive factors for lymph node metastasis (*P* = 0.004, *P* = 0.008, and *P* = 0.028, respectively). Among these factors, only low ALDH1 expression in cancer cells was significantly correlated with the farther spreading of lymph node metastasis (mediastinal lymph node, pathological N2) (*P* = 0.046) and the metastatic lymph node ratio (metastatic/resected) (*P* = 0.028). On the other hand, in the primary tumors, ALDH1 expression in the cancer cells was not associated with lymph node metastasis. Intralymphatic cancer cells expressing low ALDH1 levels exhibited lower E-cadherin expression levels than cancer cells with high levels of ALDH1 expression (*P* = 0.015).

**Conclusions:**

Intralymphatic cancer cells expressing low levels of ALDH1 and infiltrating macrophages expressing CD204 have a critical impact on lymph node metastasis. Our study also highlighted the significance of evaluating the biological properties of intralymphatic tumors for tumor metastasis.

## Introduction

Lung adenocarcinoma is the most common histological type among primary lung cancers and is one of the most frequent causes of death among cases with advanced disease [1], [2]. Lymph node (LN) metastasis is conceivably the preliminary step of distant metastasis and is directly linked with a poor prognosis [3], [4]. The process of LN metastasis starts with the intravasation of cancer cells followed by escape from anoikis in the lymphatics, and transmigration to the LNs [5], [6].

Recently, investigators studying the cancer initiating/stem cells (CIC/CSCs) hypothesis have suggested the existence of a subset of cancer cells with the ability to undergo metastasis initiation, including those characterized by a high migration potential and an ability to adapt to the metastatic site [7]. In particular, cancer cells involved in metastasis formation, which are also called metastasis-initiating cells, were identified by their close relationship with CIC/CSCs, and the elucidation of these properties was regarded as being critically important. In non-small cell lung cancer (NSCLC), the expression of CIC/CSCs-related markers, for example aldehyde dehydrogenase1 (ALDH1), octamer-binding transcription factor 4 (OCT4), NANOG, SRY-box 2 (SOX2), and Caveolin-1, were reportedly correlated with treatment resistance, and disease recurrence, and survival [8]–[11].

Recent reports have revealed that cancer-associated stromal cells present with the primary or circulating tumor cells play an important role in cancer cell survival and transmigration [12], [13]. Another study revealed that circulating stromal cells with tumor fragments promote the rapid growth of accompanying metastatic cancer cells [6].

**Figure 1:**
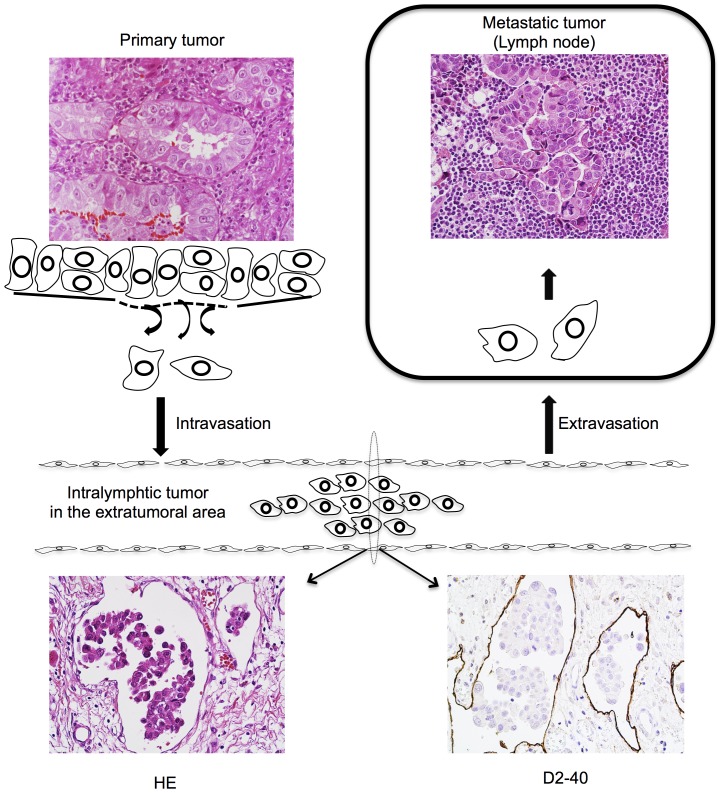
Scheme showing the process of lymphogenic metastasis.

The lymphatics within the extratumoral area have been postulated to act as conduits connecting the primary site with the metastatic LN (Figure 1). We hypothesized that the biological characteristics of intralymphatic tumor cells in the extratumoral area may be more informative than those of the primary tumors with regard to clarifying the process of cancer metastasis. The aim of the present study was to identify how the immunophenotypic features of cancer cells and infiltrating stromal cells in the extratumoral lymphatics are correlated with LN metastases.

## Materials and Methods

### Ethic Statement

Collection and the use of biopsies from lung adenocarcinoma patients were approved by the National Cancer Center Institutional Review Board (approval number: 2013-026). The written informed consent was obtained from all patients involved in the study.

### Patients

A total of 2087 consecutive adenocarcinoma patients underwent complete resections involving a lobectomy or more extensive procedures and systematic LN dissections between May 1998 and December 2012 at the National Cancer Center Hospital East. Patients who received preoperative therapy (chemotherapy or thoracic radiotherapy), who underwent a limited surgery (segmentectomy or wedge resection), or in whom a mediastinal lymphadenectomy was not performed were excluded. Among the remaining cases, we selected those that had been diagnosed as having lymphatic permeation in the extratumoral area according to previously reported criteria [14]. A total of 127 cases were selected, but 20 cases with poor-quality specimens were subsequently excluded. The remaining 107 cases were included in the present study. The clinicopathological features of the cases were collected from the clinical records.

We defined a pN1 state as the involvement of an ipsilateral intrapulmonary, peribronchial, or hilar LN metastasis and a pN2 state as the involvement of a mediastinal or subcranial LN metastasis according to the 7^th^ edition of the TNM classification. The percentage of metastatic LNs was calculated by dividing the number of metastatic LNs by the number of dissected LNs and multiplying by 100. The median number of dissected LNs was 14 nodules.

### Histopathological Studies

Surgical specimens were fixed in 10% formalin or methanol and were embedded in paraffin. The specimens were then sectioned (4 µm) and stained using hematoxylin and eosin. Vascular invasion and pleural invasion were also evaluated using Verhoeff-Van Gieson staining.

The histologic diagnoses were based on the fourth-revised World Health Organization's histologic classification. The disease stages were based on the 7^th^ edition of the TNM classification. All extratumoral lymphatic permeations were confirmed by immunostaining with anti D2-40 antibody. The median number of evaluated lymphatic vessels that contained tumor tissue was 7.

### Immunohistochemistry

The markers used in this study were ALDH1 (clone 44ALDH; BD Bioscience, San Jose, CA, USA), OCT4 (clone 4H2; Applied Biological Materials, Richmond, BC, Canada), NANOG (clone 2C4; Applied Biological Materials), SOX2 (clone 3A2; Applied Biological Materials), Caveolin-1 (clone D46G3; Cell Signaling Technology, Danvers, MA, USA), α-Smooth muscle actin (α-SMA; clone 1A4; DAKO, Glostrup, Denmark), CD34 (clone QBENT/10; Acris antibodies, San Diego, CA, USA), CD204 (Scavenger Receptor class A-E5; Trans Genic Hyogo, Japan), and E-cadherin (clone NCH-38; DAKO). The slides were deparaffinized with xylene and rehydrated in a graded ethanol series. For antigen retrieval, the sections were placed in citrate buffer and were heated at 95°C for 20 minutes. After the inhibition of endogenous peroxidase activity, individual slides were incubated overnight at 4°C with primary antibodies. The slides were washed with phosphate-buffered saline and then incubated with EnVision (DAKO) for 1 hour at room temperature, and the color reaction was developed in 2% 3, 3′-diaminobenzidine in 50 mM Tris buffer (pH 7.6) containing 0.3% hydrogen peroxidase. Finally, the sections were counterstained with Meyer hematoxylin, dehydrated, and mounted. Omission of primary antibody served as a negative control in each marker and we confirmed there was no positive product. The normal bronchial epithelial cells (in ALDH1 and SOX2), alveolar macrophages (in CD204 and ALDH1) were used as internal positive controls.

### Evaluation of immunohistochemistry

**Figure 2:**
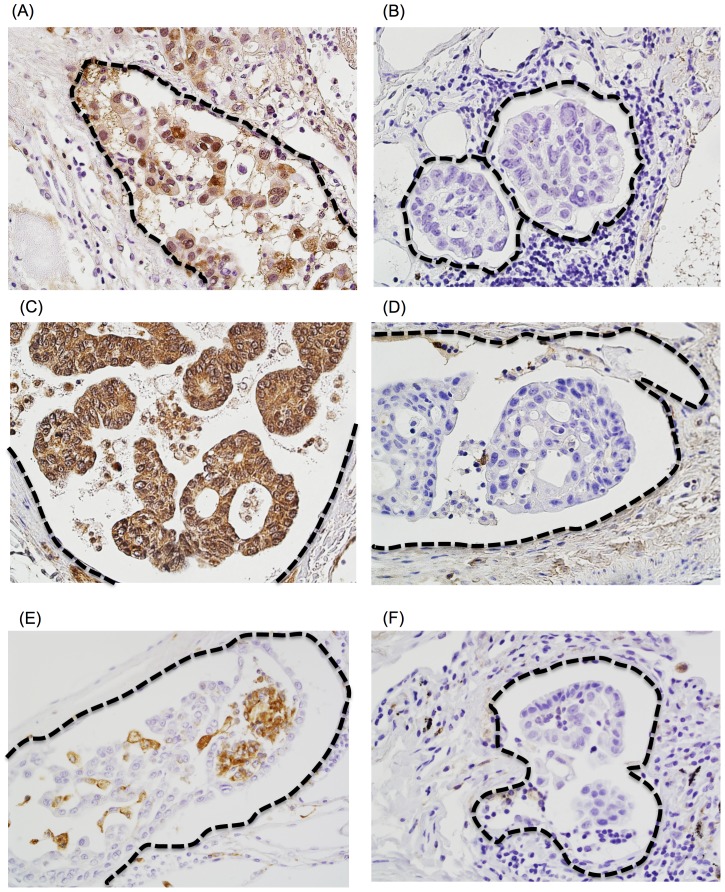
Immunohistochemical staining of intralymphatic cancer cells (A, B, C, D) and stromal cells (E, F). (A) high ALDH1 expression level, (B) low ALDH1 expression level, (C) high SOX2 expression level, (D) low SOX2 expression level, (E) higher number of CD204-positive macrophages, (F) lower number of CD204-positive macrophages.

Two pathologists (K.K. and G.I.) who had no knowledge of the patient's clinicopathological data evaluated the individual sections under a light microscope. The labeling scores for the cancer cells were calculated by multiplying the percentage of positive cancer cells per each lesion (0%–100%) by the staining intensity level (0 =  negative; 1 =  weak; 2 =  strong). As for ALDH1, the nuclear and cytoplasmic expressions were judged as positive. OCT4, NANOG, and SOX2 expression were judged as positive when nuclear expression was observed. On the other hand, Caveolin-1 and E-cadherin expressions were positive when the cellular membrane and cytoplasmic expressions were observed (Figure 2A–E, I). The cut-off values were defined as the median staining score. For α-SMA, CD34 and CD204, the number of positive infiltrating cells were counted under a microscope at ×400 (area  = 0.0625 mm^2^; Figure 2G–I). The cut-off values were defined as the median number of positive infiltrating cells. The inter-observer variability of staining evaluation was very little differentiation and the concordance was high (Cohen's kappa coefficients stayed with in 0.88 to 1.00 in each evaluated proteins).

### Statistical Analysis

The significance of differences between two groups was evaluated using the Fisher exact test, a multinomial logistic regression analysis, or the Student t-test. All the reported *P*-values were two sided, and the significance level was set at <0.05. The statistical analyses were performed using JMP® for Macintosh, version 9.0 (SAS Institute Inc., Cary, NC, USA).

## Results

### Relationship between clinicopathological characteristics and lymph node metastasis

Ipsilateral intrathoracic LN metastases were detected in specimens from 86 patients (80%). Intrapulmonary metastases were occasionally detected in resected specimens from 35 patients (33%). The disease-free survival and overall survival periods were significantly shorter in the group with lymph node metastasis (*P* = 0.010 and *P* = 0.018, respectively) (Figure S1).

A univariate analysis revealed that only intrapulmonary metastasis was significantly associated with LN metastasis. Sex, age, smoking history, tumor size, vascular invasion, and pleural invasion were not significantly associated with LN metastasis in this cohort (Table 1).

**Table 1:** Relationship between clinicopathological characteristics and lymph node metastasis.

Category	Subcategory	LN meta. (+) N = 86	LN meta. (−) N = 21	p-value
Sex	Male	61	13	0.438
	Female	25	8	
Age, year	Median (range)	67 (41–86)		
	≥70	32	8	1
	70>	54	13	
Smoking	Ex or current	59	13	0.608
	Never	27	8	
Tumor size, cm	≥3.0	38	12	0.286
	3.0>	48	9	
Histology	Mixed subtype	77	20	0.899
	Others **	9	1	
Vascular invasion	Positive	65	15	0.78
	Negative	21	6	
Pleural invasion	Positive	48	13	0.806
	Negative	38	8	
Pulmonary metastasis	Positive	34	1	0.002*
	Negative	52	20	

*Considered to be statistically significant (p<0.05).

**Solid adenocarcinoma with mucin.

### Univariate and multivariate analyses of cancer cells and stromal cells for the presence of LN metastasis

The median staining scores (cut-off value) were 0 for ALDH1, 90 for OCT4, 20 for NANOG, 30 for SOX2, and 0 for Caveolin-1, respectively. In univariate analyses, a low ALDH1 expression (*P* = 0.004) and a high SOX2 expression (*P* = 0.008) in cancer cells were significantly correlated with LN metastasis. However, no significant correlations were observed between LN metastasis and the expression levels of OCT4, NANOG, or Caveolin-1 (Table 2).

**Table 2:** Univariate analysis about relationship of immunohistochemical staining of intralymphatic tumor and LN metastasis (Cancer cells).

Antibodies	Score	LN meta. (+) N = 21	LN meta. (−) N = 86	p-value, univariate	Odds ratio (95%CI)	p-value, multivariate
ALDH1	high	13	23	*0.004	low/high, 3.25 (1.11–9.28)	*0.031
	low	8	63			
OCT4	high	8	46	0.232		
	low	13	40			
NANOG	high	10	53	0.323		
	low	11	33			
SOX2	high	6	53	*0.008	high/low, 4.09 (1.38–13.4)	*0.011
	low	15	33			
Caveolin-1	high	1	12	0.456		
	low	20	74			

*Considered to be statistically significant (p<0.05).

Table 3 shows the relationship between infiltrating stromal cells and LN metastasis. The median number of CD204(+) macrophages was 4.5 (cells/0.625 mm^2^). However, the number of cases with CD34(+) endothelial cells and α-SMA(+) myofibroblasts were 9 and 7 cases, respectively. In the univariate analyses, only a high number of intralymphatic CD204(+) macrophages was significantly correlated with LN metastasis (*P* = 0.023).

**Table 3:** Univariate analysis about relationship of immunohistochemical staining of intralymphatic tumor and LN metastasis (Infiltrating stromal cells).

Antibodies	Score	LN meta. (+) N = 21	LN meta. (−) N = 86	p-value, univariate	Odds ratio (95%CI)	p-value, multivariate
SMA	high	1	6	1		
	low	20	80			
CD34	high	1	8	0.685		
	low	20	78			
CD204	high	6	49	*0.028	low/high, 3.45 (1.16–11.4)	0.026
	low	15	37			

*Considered to be statistically significant (p<0.05).

Multivariate logistic regression models were used to determine the independent factors affecting LN metastasis. A low ALDH1 expression, high SOX2 expression, and a higher number of CD204(+) macrophages were independent predictive factors for LN metastasis (odds ratio [95%CI] = 3.25 [1.11 – 9.82], *P* = 0.031 for ALDH1; 4.09 [1.38 – 13.4], *P* = 0.011 for SOX2; and 3.45 [1.16 – 11.4], *P* = 0.026 for CD204(+) macrophages).

We also evaluated the relationship between these factors and LN metastasis in the primary tumor (Table 4). However, only a high SOX2 expression level in the cancer cells within the primary tumor was significantly correlated with LN metastasis (p = 0.008); ALDH1 expression in the cancer cells and the number of CD204(+) macrophages were not correlated with LN metastasis (*P* = 0.230 and *P* = 0.088, respectively). Relationship between other clinicopathological characteristics and immunohistochemical staining in primary tumors were shown in Table S1–S3.

**Table 4:** Univariate analysis about relationship of immunohistochemical staining of primary tumor and LN metastasis.

Antibodies	Score	LN meta. (+) N = 21	LN meta. (−) N = 86	p-value, univariate
ALDH1	high	14	44	0.23
	low	7	42	
SOX2	high	6	51	*0.008
	low	15	35	
CD204	high	7	48	0.088
	low	14	38	

*Considered to be statistically significant (p<0.05).

### Correlation of ALDH1, SOX2 expression in cancer cells and the number of CD204(+) macrophages with the aggressiveness of LN metastasis

We investigated the expansion of thoracic LN metastasis based on the pN1 and pN2 classification in patients with LN metastasis. pN2 disease, which is characterized by the more distant spreading of LN metastasis, is known to represent a higher degree of malignancy in lung adenocarcinomas [4].

A total of 20 patients were diagnosed as pN1, and 66 patients were diagnosed as pN2. A low ALDH1 expression level was significantly more frequent among the pN2 patients (*P* = 0.046); however, the high expression of SOX2 and a higher number of CD204(+) macrophages were not associated with a pN2 diagnosis (*P* = 0.440 and 0.121, respectively)(Table 5).

**Table 5:** The aggressiveness of lymph node metastasis (Pathological N stage).

Antibodies	Score	pN1: N = 20	pN2: N = 66	p-value, univariate
ALDH1	high	9	14	*0.046
	low	11	52	
SOX2	high	14	39	0.44
	low	6	27	
CD204	high	8	41	0.121
	low	12	25	

*Considered to be statistically significant (p<0.05).

The metastatic LN ratio can also reflect the aggressiveness of metastasis [15]–[18]. A low ALDH1 expression level was significantly correlated with a high percentage of metastasis (*P* = 0.015), but the expression of SOX2 and a higher number of CD204(+) macrophages were not (*P* = 0.372 and 0.054, respectively) (Table 6). Based on the above findings, a low ALDH1 level in cancer cells was suggested to have the strongest impact on lymphogenic metastasis.

**Table 6:** The aggressiveness of lymph node metastasis (Metastatic lymph node ratio).

Antibodies	Score	Percentage of metastatic LN (%) Mean, (±SE)	p-value, univariate
ALDH1	high	23.0 (±4.7)	*0.015
	low	37.2 (±3.3)	
SOX2	high	34.6 (±3,7)	0.372
	low	29.6 (±4.2)	
CD204	high	37.6 (±3.8)	0.054
	low	26.9 (±3.9)	

*Considered to be statistically significant (p<0.05).

### Correlation between ALDH1 expression and E-cadherin expression in intralymphatic cancer cells

Recent studies have linked the epithelial-mesenchymal transition (EMT) process to the induction of metastasis-initiating features [19], [20]. We analyzed the expression of E-cadherin in the cancer cells and its association with the ALDH1 expression status.

**Figure 3:**
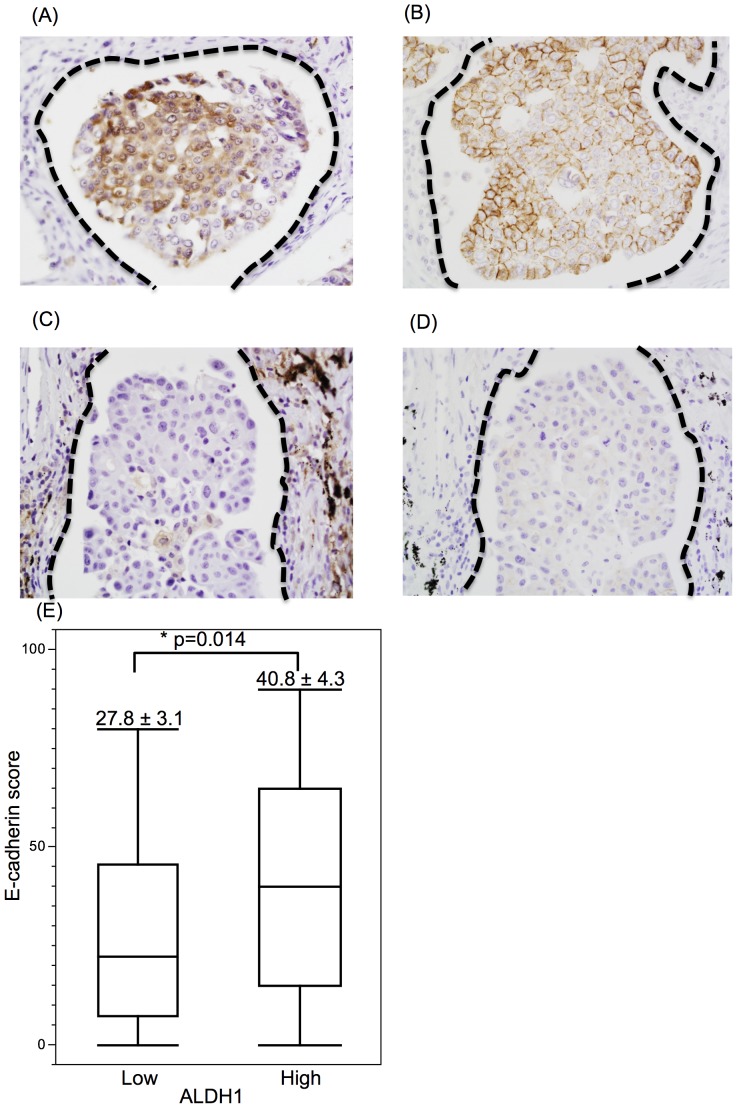
Relationship between ALDH1 and E-cadherin expression. (A) intralymphatic cancer cells showing a high level of ALDH1 expression, and (B) intralymphatic cancer cells showing a high level of E-cadherin expression. The images shown in A and B were obtained from the same case. (C) Intralymphatic cancer cells with a low level of ALDH1 expression. (D) Intralymphatic cancer cells with a low level of E-cadherin expression. The images shown in C and D were also obtained from the same case. (E) Comparison of E-cadherin expression between cancer cells with low and high levels of ALDH1 expression.

As shown in Figure 3, the cancer cells in the low ALDH1 expression group had a significantly lower E-cadherin score (mean ± SE, 27.8±3.1 for low ALDH1 group and 40.8±4.3 for high ALDH1 group, *P* = 0.014). The expressions of other CIC/CSCs related markers, including OCT4, NANOG, SOX2, and Caveolin-1, were not correlated with the E-cadherin score (Figure S2).

## Discussion

Since tumors in the extratumoral lymphatics are considered to represent the preceding phase of LN metastasis, examining the biological characteristics of these cells is likely to be important for elucidating the mechanism of lymphogenic metastases. The current study demonstrated that the low expression of ALDH1 in cancer cells within the lymphatics was an independent predictive factor of LN metastasis. Furthermore, low ALDH1 expression impacts metastatic aggressiveness, including pN2 disease with farther spreading and the metastatic LN ratio. We also found that a higher number of CD204(+) macrophages in the extratumoral lymphatics was associated with LN metastasis. The results of this study suggest the possibility that the microenvironment of the tumor tissue created by intralymphatic cancer cells and stromal cells in the extratumoral area has a considerable impact on LN metastasis.

We performed survival analysis, however, only intralymphatic SOX2 expression significantly associated with overall survival, but intralymphatic ALDH1 and CD204 positive macrophages did not. Because this study population included the cases performed operation until quite recently, we considered the reason of those results was affected by insufficient follow up time and many censored cases.

The prognostic value of ALDH1 expression in cancer cells in the primary lesion is controversial. A high ALDH1 expression level has been used as a biomarker predicting a poor prognosis in breast cancer, serous ovarian cancer, colorectal cancer, and several other tumors [21]–[23]. In contrast, Dimou et al. noted that the expression of ALDH1 was independently associated with a better prognosis in patients with NSCLC, especially those with adenocarcinoma. In melanoma cells, both ALDH-positive and ALDH-negative cells have exhibited similarly high clonal formation abilities *in vitro* and cancer initiation abilities *in vivo* when isolated from melanoma xenografts [24]. Okudela et al. demonstrated that the forced expression of ALDH1A1 in a NSCLC cell line remarkably reduced clonogenicity and prolonged the doubling time *in vivo* [25], and these results are compatible to those obtained in the present study. To clarify the role of ALDH1 in intralymphatic cancer cells more clearly, the *in vivo* and/or *in vitro* relevance of ALDH1 to tumorigenicity should be examined in ALDH1-sorted cancer cells.

In recent studies examining the EMT, the loss of E-cadherin expression has been shown to play a key role in the metastatic process [19], [20]. This reflection in part supports our results that a low ALDH1 expression in intralymphatic cancer cells was correlated with a low E-cadherin expression. Another noteworthy fact is that the expression of ALDH1 in cancer cells in the primary tumor was not correlated with the expression of E-cadherin (Figure S3) and was not a significant predictive factor for LN metastasis. Thus, our new findings suggest the possibility that a low ALDH1 expression level in cancer cells present only in the extratumoral lymphatics might induce the EMT, leading to the acquisition of tumorigenic capacity.

About ALDH1, downregulated cases (staining score in intralymphatics decreased less than one-half in primary tumors) significantly associated with lymph node metastasis than other cases (p = 0.012) (Table S4). Otherwise, about the group with SOX2 upregulation or increased CD204+ macrophages (staining score in intralymphatics increased more than double in primary tumors), the frequency of lymph node metastasis was almost equivalent than other cases (p = 0.440 and 0.584, respectively). It is plausible to think that deregulation of ALDH1 expression in intralymphatics tumor cells may impact on lymphogenic metastasis by occurring EMT.

Macrophage M1/M2 polarization with either pro-inflammatory or anti-inflammatory properties impact on malignant neoplasm progression and could be discriminated by immunohistochemical staining. CD204 was macrophage scavenger receptor, and thought to be expressed not only M2 macrophages but dendritic cells. We examined the concordance between number of CD204+ cells and another M2 macrophage marker using CD163. As shown in figure S4, significantly high correlation between CD204 number and CD163 number in both primary site (N = 40) and intralymphatics (N = 40) (R-value were 0.86 and 0.89, respectively), suggesting that CD204 cells that we counted were M2 macrophages but not dendritic cells.

In the current study, a higher number of intralymphatic CD204(+) macrophages was significantly correlated with LN metastasis. We previously reported that the number of CD204(+) macrophages was correlated with an intralymphatic tumor morphology consisting of multiple small nests, which tends to occur in cases with pulmonary metastasis [6]. CD204(+) macrophages reportedly exhibit a tumor-promoting function in several tumors by secreting matrix metalloproteinase (MMP)-1, MMP-3, MMP-7, MMP-9, MMP-12, vascular endothelial growth factor (VEGF), and transforming growth factor (TGF)-β [26], [27]. Taken together, intralymphatic CD204(+) macrophages may also play an important role in the early lymphogenic metastatic process through the promotion of extracellular matrix remodeling, angiogenesis, and the EMT at a secondary site [28], [29].

SOX2 has been considered as a CIC/CSCs related molecule. Xiang et al noticed that the knockdown of SOX2 in cancer cells suppressed experimental pulmonary metastases in an animal model, suggesting that cancer cells in which SOX2 expression is upregulated might be a candidate for metastasis-initiating cells. [30] Singh et al. demonstrated that SOX2 expression had a more critical impact on cancer cell-proliferation than OCT4 and NANOG [31], [32], and a high level of SOX2 expression is also known to be a prognostic factor in NSCLC [33]. Our result confirms that SOX2 also plays an important role in the progression of cancers caused by lymphogenic metastasis. Additionally, a high SOX2 expression level in primary cancer cells was also associated with LN metastasis, which differs from ALDH1 and CD204(+) macrophages in this respect. Cancer cells with high levels of SOX2 expression might also have an impact on the lymph vessel invasion process at the primary site.

In conclusion, this study showed the significance of evaluating the biological properties of intralymphatic tumors. Moreover, the microenvironment of intralymphatic tumors created by cancer cells with a low level of ALDH1 expression and a number of CD204(+) macrophages has a significant impact on LN metastasis. The current results also suggest the possibility that intralymphatic cancer cells with a low level of ALDH1 expression and CD204(+) macrophages could be useful as a new molecular target, especially as an adjuvant therapy, in patients exhibiting lymphatic permeation. Further *in vivo* investigation of the metastatic mechanism will be important.

## Supporting Information

Figure S1 **Kaplan-Meier analysis for disease-free survival and overall survival stratified according to the existence of lymph node metastasis.** The median follow-up period was 22.4 months. (A) Disease-free survival for all patients. (B) Overall survival for all patients.
(TIF)

Figure S2 **Comparison of E-cadherin expression between cancer cells with high and low levels of CIC/CSCs-related markers**. (A) OCT4, (B) NANOG, (C) SOX2, and (D) Caveolin-1.
(TIF)

Figure S3 **Comparison of E-cadherin expression between ALDH1 in primary tumor cells.**
(TIF)

Figure S4 **Relationship between CD204+ cells and CD163+ cells.** (A) Primary tumor tissue. (B) Intralymphatic tumor tissue.
(TIF)

Table S1 **Relationship between clinicopathological characteristics and ALDH1 expression (intralymphatic tumor cells).**
(DOCX)

Table S2 **Relationship between clinicopathological characteristics and SOX2 expression (intralymphatic tumor cells).**
(DOCX)

Table S3 Relationship between clinicopathological characteristics and CD204-positive macrophages expression (intralymphatic tumor cells).
(DOCX)

Table S4 **Molecular expression changes and lymph node metastasis.**
(DOCX)
