# Supplementary figures and images for: Identification of Biological Properties of Intralymphatic Tumor Related to the Development of Lymph Node Metastasis in Lung Adenocarcinoma
Source: PLoS One. 2013 Dec 23;8(12):e83537. doi: 10.1371/journal.pone.0083537 (PMC3871680; doi:10.1371/journal.pone.0083537)

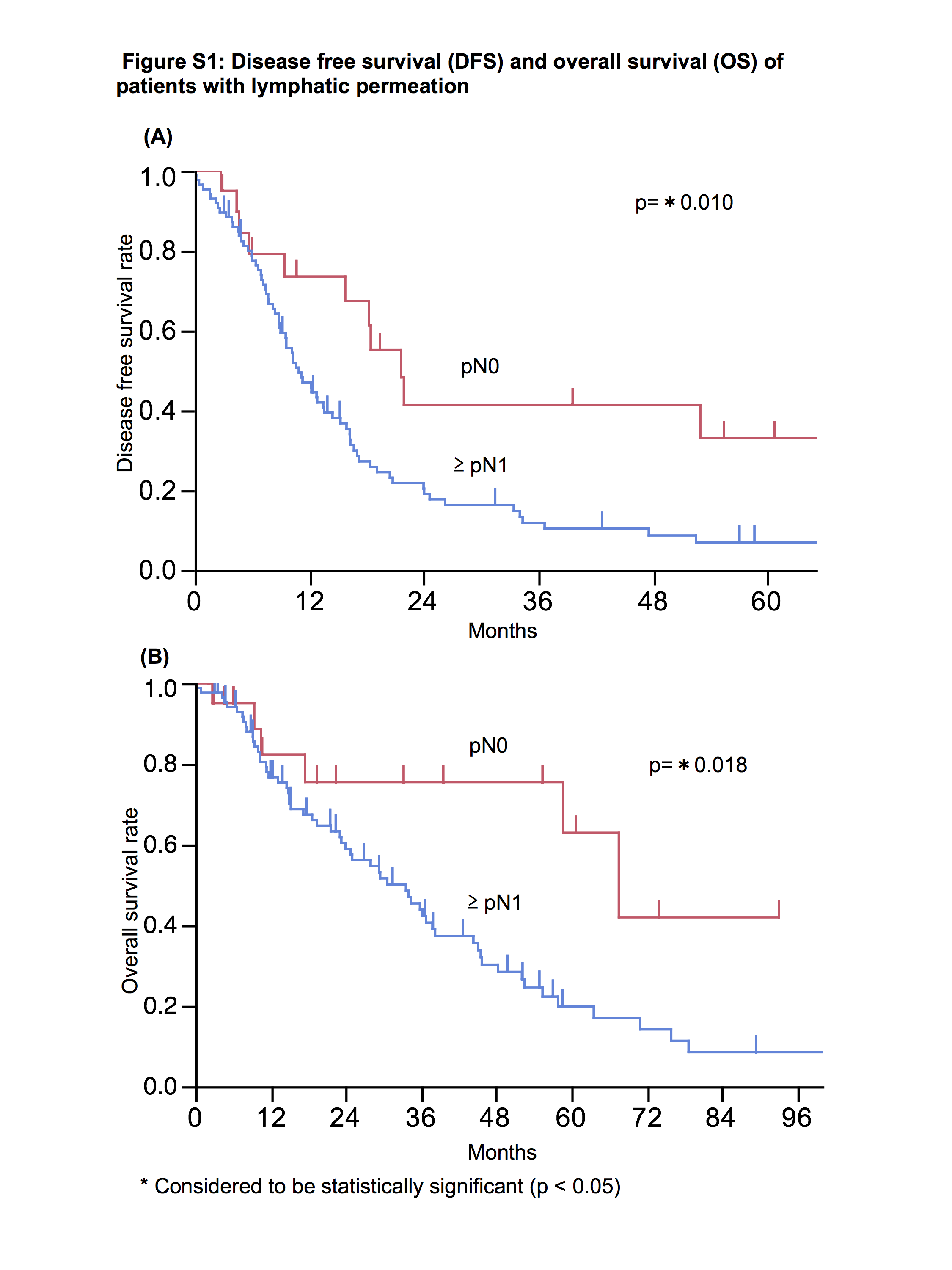

Supplement: Figure S1 — Kaplan-Meier analysis for disease-free survival and overall survival stratified according to the existence of lymph node metastasis. The median follow-up period was 22.4 months. (A) Disease-free survival for all patients. (B) Overall survival for all patients. (TIF) [file pone.0083537.s001.tif]

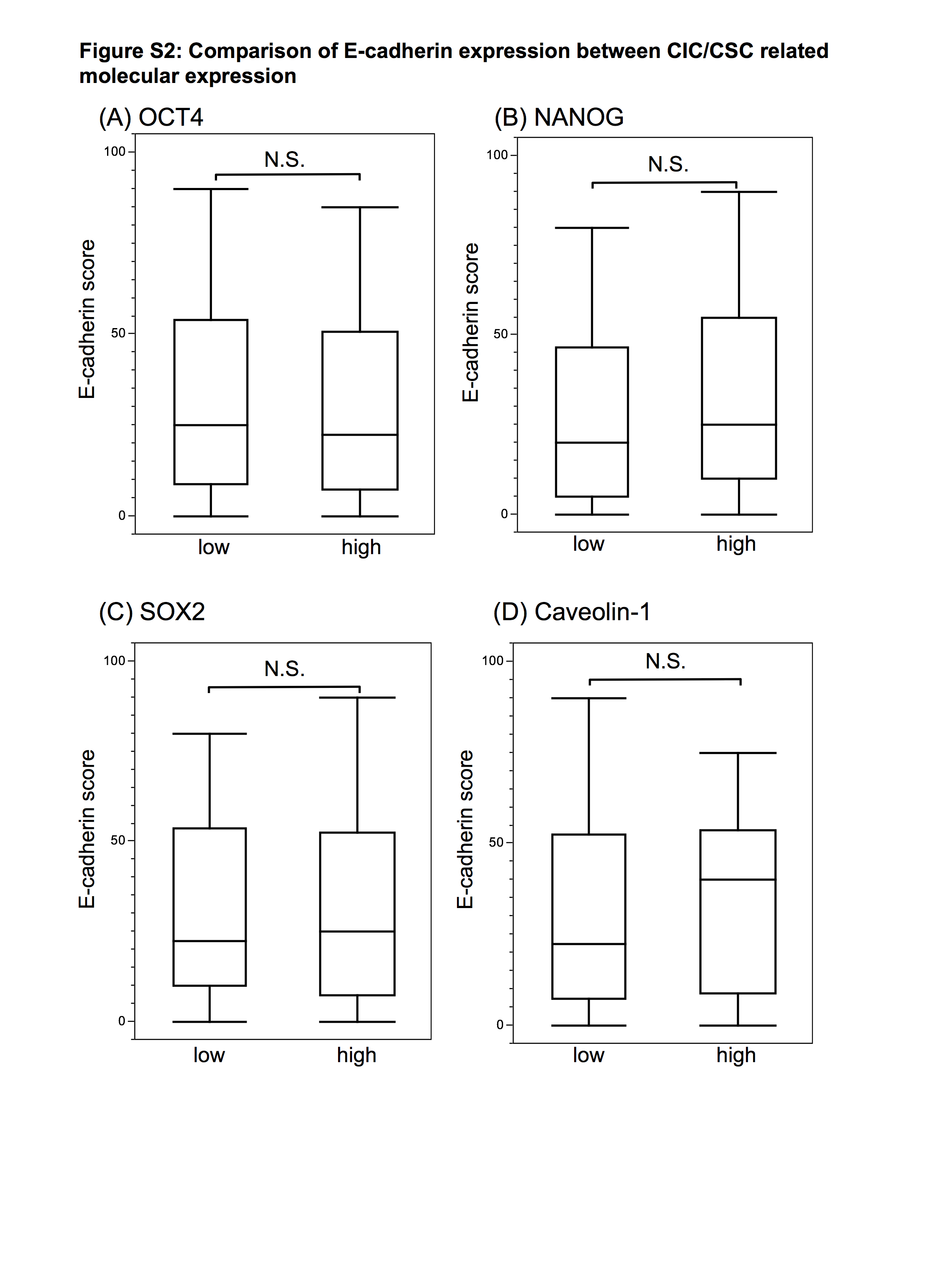

Supplement: Figure S2 — Comparison of E-cadherin expression between cancer cells with high and low levels of CIC/CSCs-related markers. (A) OCT4, (B) NANOG, (C) SOX2, and (D) Caveolin-1. (TIF) [file pone.0083537.s002.tif]

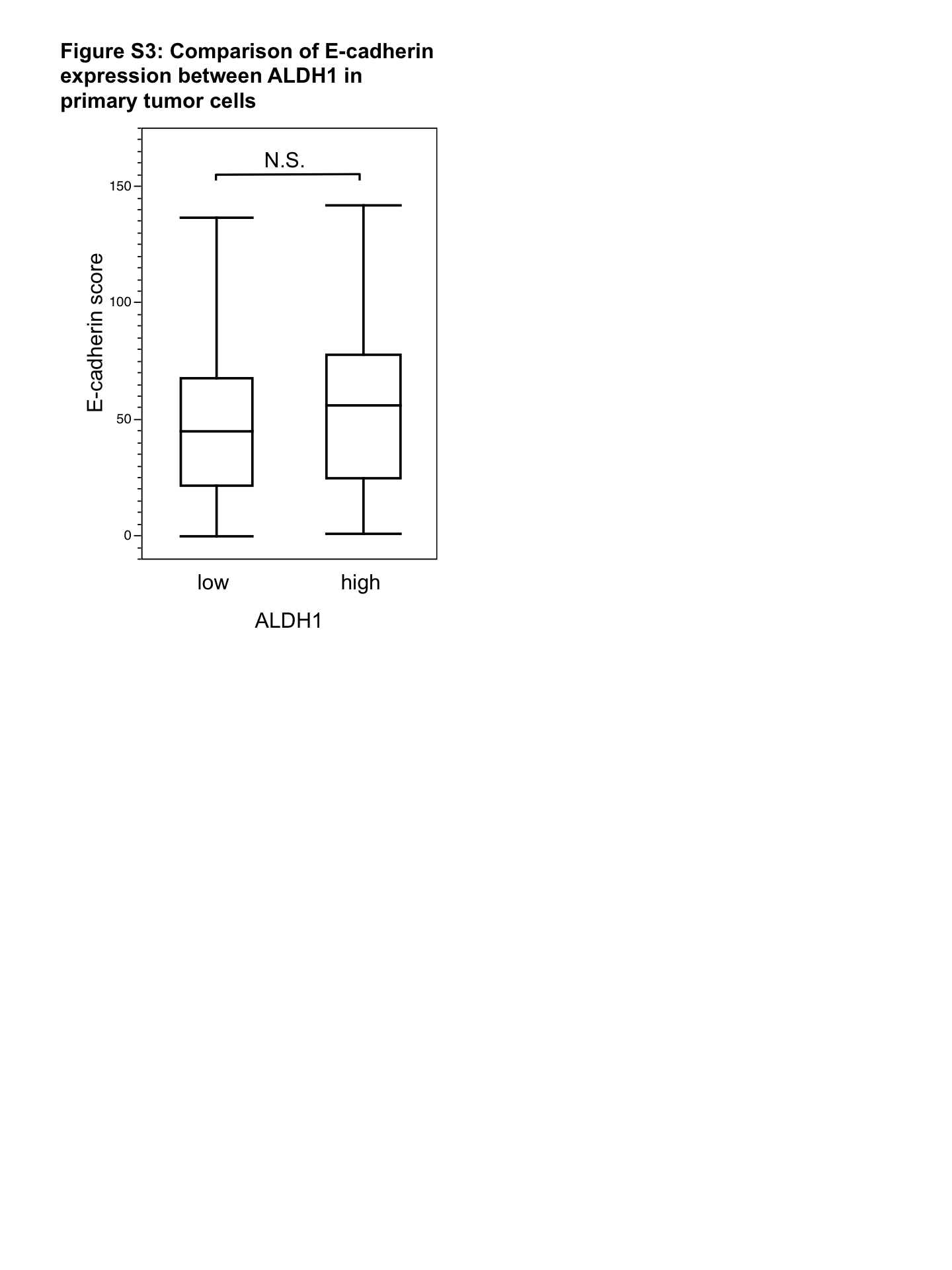

Supplement: Figure S3 — Comparison of E-cadherin expression between ALDH1 in primary tumor cells. (TIF) [file pone.0083537.s003.tif]

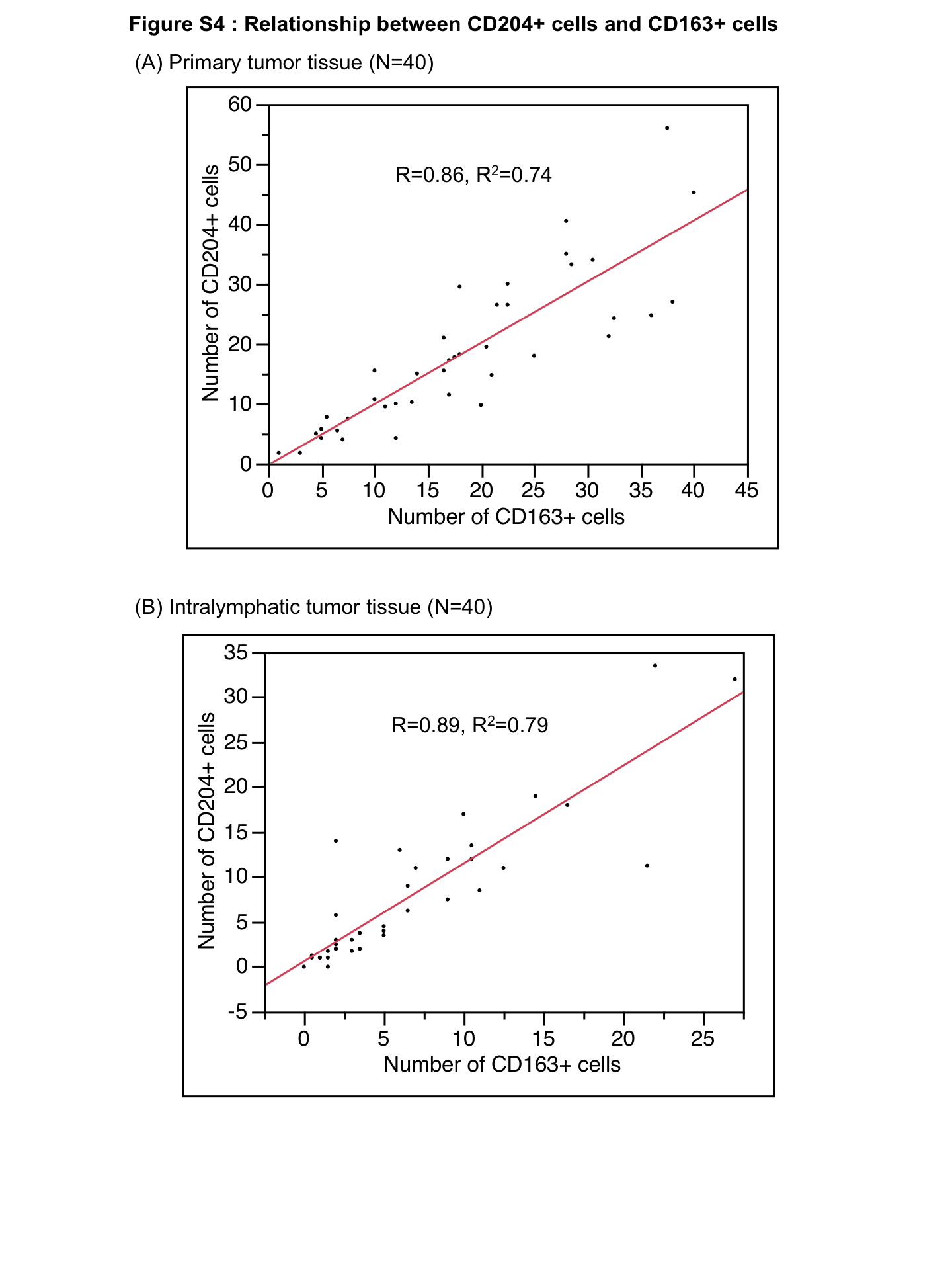

Supplement: Figure S4 — Relationship between CD204+ cells and CD163+ cells. (A) Primary tumor tissue. (B) Intralymphatic tumor tissue. (TIF) [file pone.0083537.s004.tif]
