# Supplementary material for: Identification of Biological Properties of Intralymphatic Tumor Related to the Development of Lymph Node Metastasis in Lung Adenocarcinoma
Source: PLoS One. 2013 Dec 23;8(12):e83537. doi: 10.1371/journal.pone.0083537 (PMC3871680; doi:10.1371/journal.pone.0083537)
Supplement: Table S1 — Relationship between clinicopathological characteristics and ALDH1 expression (intralymphatic tumor cells). (DOCX) [file pone.0083537.s005.docx]

Table S1. Relationship between clinicopathological characteristics and ALDH1 expression (intralymphatic tumor cells)

| Category | Subcategory | ALDH1 high (N=36) | ALDH1 low (N=71) | p-value |
| --- | --- | --- | --- | --- |
| Sex | Male | 26 | 48 | 0.665 |
|  | Female | 10 | 23 |  |
| Age, year | ≥70 | 13 | 27 | 1.000 |
|  | 70> | 23 | 44 |  |
| Smoking | Ex or current | 25 | 47 | 0.829 |
|  | Never | 11 | 24 |  |
| Tumor size, cm | ≥3.0 | 22 | 35 | 0.307 |
|  | 3.0> | 14 | 36 |  |
| Histology | Mixed subtype | 32 | 65 | 0.730 |
|  | Others * | 4 | 6 |  |
| Vascular invasion | Positive | 26 | 54 | 0.814 |
|  | Negative | 10 | 17 |  |
| Pleural invasion | Positive | 48 | 13 | 0.310 |
|  | Negative | 38 | 8 |  |
| Pulmonary metastasis | Positive | 12 | 23 | 1.000 |
|  | Negative | 24 | 48 |  |

* Solid adenocarcinoma with mucin
