# Supplementary material for: Identification of Biological Properties of Intralymphatic Tumor Related to the Development of Lymph Node Metastasis in Lung Adenocarcinoma
Source: PLoS One. 2013 Dec 23;8(12):e83537. doi: 10.1371/journal.pone.0083537 (PMC3871680; doi:10.1371/journal.pone.0083537)
Supplement: Table S2 — Relationship between clinicopathological characteristics and SOX2 expression (intralymphatic tumor cells). (DOCX) [file pone.0083537.s006.docx]

Table S2. Relationship between clinicopathological characteristics and SOX2 expression (intralymphatic tumor cells)

| Category | Subcategory | SOX2 high (N=59) | SOX2 low (N=48) | p-value |
| --- | --- | --- | --- | --- |
| Sex | Male | 42 | 32 | 0.676 |
|  | Female | 17 | 16 |  |
| Age, year | ≥70 | 20 | 20 | 0.439 |
|  | 70> | 39 | 28 |  |
| Smoking | Ex or current | 45 | 27 | 0.038* |
|  | Never | 14 | 21 |  |
| Tumor size, cm | ≥3.0 | 35 | 22 | 0.178 |
|  | 3.0> | 24 | 26 |  |
| Histology | Mixed subtype | 53 | 44 | 1.000 |
|  | Others ** | 6 | 4 |  |
| Vascular invasion | Positive | 44 | 36 | 1.000 |
|  | Negative | 15 | 12 |  |
| Pleural invasion | Positive | 34 | 27 | 1.000 |
|  | Negative | 25 | 21 |  |
| Pulmonary metastasis | Positive | 17 | 18 | 0.409 |
|  | Negative | 42 | 30 |  |

* Considered to be statistically significant (p < 0.05)

** Solid adenocarcinoma with mucin
