# Supplementary material for: Identification of Biological Properties of Intralymphatic Tumor Related to the Development of Lymph Node Metastasis in Lung Adenocarcinoma
Source: PLoS One. 2013 Dec 23;8(12):e83537. doi: 10.1371/journal.pone.0083537 (PMC3871680; doi:10.1371/journal.pone.0083537)
Supplement: Table S3 — Relationship between clinicopathological characteristics and CD204-positive macrophages expression (intralymphatic tumor cells). (DOCX) [file pone.0083537.s007.docx]

Table S3. Relationship between clinicopathological characteristics and CD204-positive macrophages expression (intralymphatic tumor cells)

| Category | Subcategory | CD204+ macrophages high (N=59) | CD204+ macrophages low (N=48) | p-value |
| --- | --- | --- | --- | --- |
| Sex | Male | 38 | 36 | 1.000 |
|  | Female | 17 | 16 |  |
| Age, year | ≥70 | 23 | 17 | 0.424 |
|  | 70> | 32 | 35 |  |
| Smoking | Ex or current | 39 | 33 | 0.537 |
|  | Never | 16 | 19 |  |
| Tumor size, cm | ≥3.0 | 27 | 30 | 0.440 |
|  | 3.0> | 28 | 22 |  |
| Histology | Mixed subtype | 50 | 47 | 1.000 |
|  | Others ** | 5 | 5 |  |
| Vascular invasion | Positive | 39 | 41 | 0.380 |
|  | Negative | 16 | 11 |  |
| Pleural invasion | Positive | 24 | 37 | 0.006* |
|  | Negative | 31 | 15 |  |
| Pulmonary metastasis | Positive | 20 | 15 | 0.419 |
|  | Negative | 35 | 37 |  |

* Considered to be statistically significant (p < 0.05)

** Solid adenocarcinoma with mucin
