# Supplementary material for: Identification of Biological Properties of Intralymphatic Tumor Related to the Development of Lymph Node Metastasis in Lung Adenocarcinoma
Source: PLoS One. 2013 Dec 23;8(12):e83537. doi: 10.1371/journal.pone.0083537 (PMC3871680; doi:10.1371/journal.pone.0083537)
Supplement: Table S4 — Molecular expression changes and lymph node metastasis. (DOCX) [file pone.0083537.s008.docx]

Table S4. Molecular expression changes and lymph node metastasis.

| Antibodies | Change of expression | LN meta. (-) N=21 | LN meta. (+) N=86 | p-value |
| --- | --- | --- | --- | --- |
| ALDH1 | Downregulated cases § | 3 (7%) | 39 (93%) | 0.012 |
|  | Others | 18 (28%) | 47(72%) |  |
| SOX2 | Upregulated cases ¶ | 5 (14%) | 30 (86%) | 0.440 |
|  | Others | 16 (22%) | 56 (78%) |  |
| CD204 | Increased cases ¶ | 0 (0%) | 4 (100%) | 0.584 |
|  | Others | 21 (20%) | 82 (80%) |  |

* Considered to be statistically significant (p < 0.05)

§ Deregulated cases and Decreased cases were defined as staining score decreased in intralymphatics less than one-half in primary site.

¶ Upregulated cases and Increased cases were defined as staining score increased in intralymphatics more than double in primary site.
